# Effects of Curdlan on 3D-Printed Meat Analogs Based on *Stropharia rugosoannulata* Mycelium and Pea Protein Isolate: Printability, Rheology, and Texture

**DOI:** 10.3390/foods15111971

**Published:** 2026-06-02

**Authors:** Xin Hu, Haijin Tang, Jingyu Wang, Xinlian Su, Lifang Zou, Baocai Xu

**Affiliations:** 1 School of Food and Biological Engineering, Hefei University of Technology, Hefei 230601, China; 2 Anhui Ecological Fermentation Engineering Research Center for Functional Fruit Beverage, Fuyang Normal University, Fuyang 236037, China

**Keywords:** *Stropharia rugosoannulata* mycelium, curdlan, food 3D printing, meat analog, rheology, texture

## Abstract

*Stropharia rugosoannulata* mycelium is a naturally fibrous and sustainable protein source for meat analogs; however, its weak gel-forming ability and poor extrudability limit its printability and structural stability. In this study, extrusion-based 3D-printable composite inks were developed using mechanically fragmented mycelium, pea protein isolate (PPI), and curdlan (CUR). The effects of mycelium and CUR concentrations on printability, rheological properties, water-holding capacity, water distribution, thermal properties, and texture were systematically evaluated. The results showed that mechanical fragmentation for 20 s effectively dispersed the mycelial aggregates while preserving the filamentous network. CUR markedly improved extrusion continuity, print accuracy, and shape fidelity after deposition. All inks exhibited shear-thinning behavior. Increasing CUR concentration enhanced apparent viscosity, storage modulus, thixotropic recovery, water-holding capacity, and thermal stability, while converting part of the immobilized water into bound water within the gel network. In addition, CUR strengthened hydrogen bonding in the composite inks. Texture profile analysis of heated meat analogs showed that hardness, springiness, cohesiveness, gumminess, chewiness, and resilience increased progressively with increasing CUR concentration. Among the tested formulations, the ink containing 50% mycelium, 5% PPI, and 6% CUR exhibited the best balance between printability, structural stability, and meat-like texture, showing the closest textural similarity to boiled chicken breast. These findings provide a practical strategy for fabricating mycelium-based meat analogs with improved printability and meat-like texture.

## 1. Introduction

As global demand for meat continues to grow, the search for suitable protein alternatives is gaining increasing attention [1]. Among various alternative protein materials, edible fungal mycelium has attracted increasing attention owing to its rapid growth, low environmental burden, and naturally filamentous morphology [2]. Nutritionally, fungal mycelium is widely recognized for its nutritional profile. It is naturally low in fat and cholesterol, rich in various essential amino acids, alongside abundant dietary fiber, vitamins, and minerals [3]. Unlike many plant proteins, which usually require intensive structuring processes to generate fibrous textures, mushroom mycelium inherently forms a three-dimensional hyphal network resembling the fibrous architecture of muscle tissue [4]. This structural feature makes mycelium a promising raw material for producing meat analogs with improved textural realism.

Conventional methods for meat analog production, such as high-moisture extrusion, are effective at creating bulk fibrous textures but often struggle to control macroscopic shapes and the precise spatial distribution of different ingredients, such as fat and lean meat [4]. Because of its precise control over structural organization, 3D printing technology offers unique advantages in shape customization, texture optimization, and nutritional fortification of meat analogs, and has therefore become an important tool in meat analog research [5]. This technology enables the accurate reproduction of both the macroscopic shape and fibrous architecture of conventional meat [6]. Shi et al. [7] used computer-aided design modeling combined with extrusion-based 3D printing to fabricate soybean protein isolate-based analogs with structures resembling those of real fish meat; the printed products exhibited hardness and elasticity comparable to those of natural fish meat. Through layer-by-layer deposition, 3D printing can also create regions with different densities within meat analogs, thereby mimicking muscle fiber patterns and fat distribution and enhancing the layered mouthfeel during mastication [8]. Furthermore, with the continuous advancement of automated multi-nozzle systems, 3D printing presents significant potential for the scalable production of highly customized meat alternatives. For fungal protein-based meat analogs, 3D printing may help improve the regularity of fibrous structures by controlling the printing path to guide the directional alignment of mycelia, which contributes to a more meat-like texture [9].

*Stropharia rugosoannulata* is an edible mushroom with high nutritional value and favorable cultivation potential. Its mycelium can be produced by submerged fermentation, providing a controllable and scalable route for fungal biomass production [10]. However, despite its inherent fibrous morphology at the microscopic level, the direct use of mycelium in structured meat analogs remains challenging. During submerged fermentation, fluid shear forces and hyphal entanglement cause the filamentous mycelium to self-assemble into dense pellets. Consequently, the fresh harvested mycelium typically exhibits a macroscopic spherical or granular morphology [11], has weak gel-forming ability, and is difficult to extrude through the nozzle during 3D printing.

Hydrocolloids are widely used to modulate the rheological and textural properties of food gels. Curdlan (CUR), a linear β-1,3-glucan produced by microbial fermentation, is particularly attractive because of its unique thermally induced gelation behavior [12]. Upon heating, CUR forms a strong thermo-irreversible gel with high water-holding capacity and mechanical stability [13]. Li et al. [14] found that CUR can enhance the gel strength, water retention, and viscoelasticity of protein-based food systems through molecular entanglement, hydrogen bonding, and network reinforcement. Therefore, incorporating CUR into mycelium-based formulations may be an effective strategy to compensate for the weak gelation of mycelium and improve the processability of fungal protein-based meat analogs.

In this study, *S. rugosoannulata* mycelium was used as the main fibrous ingredient. PPI was incorporated as a supplementary protein source due to its low allergenicity and complementary amino acid profile [15]. Functionally, PPI exhibits water-holding and gelling properties [16], which can synergistically interact with polysaccharides and mycelial fibers to enhance the rheological properties and structural stability of extrusion-based inks. Furthermore, CUR was introduced as a thermo-irreversible gelling polysaccharide to construct printable mycelium–PPI–CUR composite inks. First, the effect of mechanical fragmentation on the morphology and dispersion of mycelial fibers was investigated to obtain a suitable fibrous structure for extrusion-based 3D printing. Subsequently, the effects of mycelium content and CUR concentration on printability, print accuracy, rheological properties, water-holding capacity, water distribution, molecular interactions, thermal stability, and texture of the printed products were systematically evaluated. Finally, the textural similarity between the optimized mycelium-based meat analog and common cooked meats was compared. This study aims to establish a feasible formulation strategy for 3D-printed fungal protein-based meat analogs and to clarify the role of CUR in improving the printability, structural stability, and meat-like texture of mycelium composite gels.

## 2. Materials and Methods

### 2.1. Materials

CUR was purchased from Shanghai Yuanye Bio-Technology Co., Ltd. (Shanghai, China). Pea protein isolate (PPI; protein content ≥ 96%) was obtained from Xi’an Xitai Biotechnology Co., Ltd. (Xi’an, China). Potato dextrose broth was purchased from Huankai Microbial Technology Co., Ltd. (Guangzhou, Guangdong, China). *S. rugosoannulata* mycelium was cultivated in our laboratory and harvested after submerged fermentation.

### 2.2. Preparation of S. rugosoannulata Mycelium

#### 2.2.1. Mycelium Cultivation and Nucleic Acid Reduction

The *S. rugosoannulata* strain (5 pieces of 8 mm mycelium agar blocks) was cultured in 100 mL of potato dextrose broth at 25 °C and 150 rpm for 7 d. The mycelium was then homogenized (8 taps/s, 3 min; Scientz09, Scientz, Ningbo, China) and used to inoculate a new PDB medium (5% (*v*/*v*) inoculum) for a second 7 d cultivation under the same conditions [17]. The harvested mycelium was subjected to nucleic acid reduction according to the method described by Nian et al. [18]. Specifically, the harvested mycelium was heated in a 68 °C water bath for 30 min, followed by filtration to remove the culture medium. The mycelium was washed three times with deionized water and stored at 4–8 °C for further use.

#### 2.2.2. Mechanical Fragmentation of Mycelium

The nucleic acid-reduced mycelium was mixed with deionized water at a mass ratio of 1:1 and homogenized using a blender (DJ12X-D135, Joyoung, Hangzhou, China) at 10,000 rpm for 10, 20, 30, and 40 s, respectively. The fragmented mycelial suspension was filtered through a 200-mesh nylon filter bag, and the retained wet mycelium was collected. The moisture content of the collected mycelium was adjusted to 75 ± 1% by adding deionized water or by mild dewatering.

### 2.3. Characterization of Fragmented Mycelium

Fragmented mycelial suspensions were evenly spread in Petri dishes and photographed using a digital camera to visually assess particle size distribution, uniformity, and dispersion state. The fragmented mycelial samples were freeze-dried and mounted on conductive adhesive tape. After gold sputter coating for 60 s, the samples were observed under a scanning electron microscope [19] (SEM, EM-30+, Coxem, Daejeon, South Korea).

### 2.4. Preparation of Mycelium–PPI–CUR Composite Inks

Composite inks were prepared according to the method of Muhedaner et al. [9] with slight modifications. The formulations are shown in Table 1. CUR powder at different concentrations, namely 0%, 2%, 4%, and 6% *w*/*w*, was dispersed in the corresponding amount of deionized water and stirred in a 55 °C water bath until a homogeneous suspension was obtained. Subsequently, PPI at 5% and mycelium at 40%, 50%, or 60% were added. The mixture was homogenized at 500 rpm for 30 min to ensure complete hydration and uniform dispersion. The prepared inks were stored at 4–8 °C prior to 3D printing and subsequent physicochemical analyses.

### 2.5. Rheological Measurements

The rheological properties of the composite inks were measured using a rotational rheometer (MCR 302, Anton Paar, Styria, Austria) equipped with a PP25 parallel plate (diameter of 25 mm and a gap of 1 mm). Before testing, the samples were equilibrated at 25 °C for 120 s. Apparent viscosity was measured over a shear rate range of 0.01–100 s^−1^. Strain sweep tests were performed at 1 Hz over a strain range of 0.01–100% to determine the linear viscoelastic region (LVR) and yielding behavior [20]. Frequency sweep tests were conducted at 0.2% strain, which was within the LVR, over a frequency range of 0.1–10 Hz. The storage modulus (G′), loss modulus (G″), and tan δ were recorded [6]. A three-interval thixotropy test (3ITT) was performed to evaluate structural recovery under simulated extrusion conditions. The test consisted of three intervals: 0.1% strain for 120 s, 100% strain for 100 s, and 0.1% strain for 180 s at 1 Hz. The recovery rate was calculated as the ratio of the recovered G′ after the third interval to the initial G′ before high-strain disruption [21].

### 2.6. 3D Printing Performance

A food 3D printer (FOODBOT-EMCN, Hangzhou Shiyin Technology Co., Ltd., Hangzhou, China) was utilized to fabricate the meat analogs. Printability was evaluated based on extrusion continuity, filament uniformity, self-supporting ability, and shape fidelity after deposition. A cuboid model with dimensions of 30 × 20 × 12 mm^3^ was printed using a 0.84 mm nozzle diameter, a layer height of 0.80 mm, and an infill density of 70%. The printing speed was 15 mm/s, and both the extrusion temperature and the print bed temperature were set to 25 °C. After printing, the samples were photographed from the front, side, and top views [22].

The dimensional deviation rates and print accuracy of the printed samples were evaluated according to Chen et al. [23] with slight modifications. The actual dimensions of the printed samples along the *X*-axis, *Y*-axis, and *Z*-axis were measured using a digital vernier caliper (resolution of 0.01 mm) within 2 min. The dimensional deviation rates along the X, Y, and Z axes were calculated using Equation (1), and the overall print accuracy was calculated using Equation (2):

(1) $$Deviation\;rates\left(\%\right)=\frac{\left|L_{s}-L_{m}\right|}{L_{m}}\times 100$$

where *L_S_* represents the measured length of the printed sample in any of the X, Y, or Z directions (mm), and *L_m_* represents the specified length of the model in the corresponding direction (mm).

(2) $$\mathrm{Print}\;\mathrm{accuracy}\;(\%)=\frac{\left(1-\frac{\left|X_{s}-X_{m}\right|}{X_{m}}\right)+\left(1-\frac{\left|Y_{s}-Y_{m}\right|}{Y_{m}}\right)+\left(1-\frac{\left|Z_{s}-Z_{m}\right|}{Z_{m}}\right)}{3}\times 100$$

where *X_S_*, *Y_S_*, and *Z_S_* are the measured dimensions of the printed sample (mm); *X_m_*, *Y_m_*, and *Z_m_* are the corresponding designed model dimensions (mm).

### 2.7. Water Holding Capacity (WHC)

WHC was determined using a centrifugation method [24]. Composite inks were weighed before centrifugation (W_0_), centrifuged at 5000× *g* and 4 °C for 10 min, and then weighed again after removal of surface water (W_1_). WHC was calculated as follows:

(3) WHC (%) = W_1_/W_0_ × 100

### 2.8. Low-Field Nuclear Magnetic Resonance (LF-NMR)

The water status and distribution of the composite inks were analyzed using LF-NMR (NMI20-060H-I, Suzhou Niumag Analytical Instrument Corporation, Suzhou, China). The measurement parameters were as follows: proton resonance frequency, 20 MHz; echo time, 0.4 ms; waiting time, 8000 ms; number of echoes, 15,000; and number of scans, 8. The transverse relaxation time (T_2_) distribution was obtained, and the relative proportions of peak areas were calculated.

### 2.9. Fourier Transform Infrared Spectroscopy (FT-IR)

The FT-IR analysis of freeze-dried composite inks was conducted using a spectrometer (Frontier, PerkinElmer, Waltham, MA, USA). The spectra were measured over the range 4000 cm^−1^ to 650 cm^−1^ with a resolution of 4 cm^−1^ and 32 scans [25].

### 2.10. Differential Scanning Calorimetry (DSC)

The thermal properties of the composite inks were analyzed using DSC (Q2000, TA Instruments, New Castle, DE, USA). Approximately 5–10 mg of each freeze-dried sample was sealed in an aluminum pan, and an empty aluminum pan was used as the reference. Samples were heated from 25 °C to 300 °C at a rate of 10 °C/min under a nitrogen atmosphere. The thermal transition behavior and heat flow curves were recorded [26].

### 2.11. Preparation of 3D-Printed Meat Analogs

The 3D-printed samples were steamed at 80 °C for 30 min in a steam oven (B9, CASDON, Shenzhen, China), cooled to room temperature, and stored at 4–8 °C before further analysis.

### 2.12. Texture Profile Analysis (TPA)

The textural properties of the 3D-printed meat analogs were measured using a texture analyzer (TA-XT Plus, Stable Micro Systems, Godalming, UK) equipped with a P/36R cylindrical probe. The test parameters were as follows: pre-test speed 2.0 mm/s, test speed 1.0 mm/s, post-test speed 2.0 mm/s, compression ratio 50%, trigger force 5 g, and interval time 5 s. Hardness, springiness, cohesiveness, gumminess, chewiness, and resilience were calculated from the force–time curves [27].

### 2.13. Statistical Analysis

All experiments were performed in triplicate. Statistical analyses were performed using SPSS 26.0. Significant differences among groups were determined by one-way analysis of variance followed by Duncan’s multiple range test, with *p* < 0.05 considered statistically significant.

## 3. Results and Discussion

### 3.1. Morphological Characterization of Fragmented Mycelium

**Figure 1:**
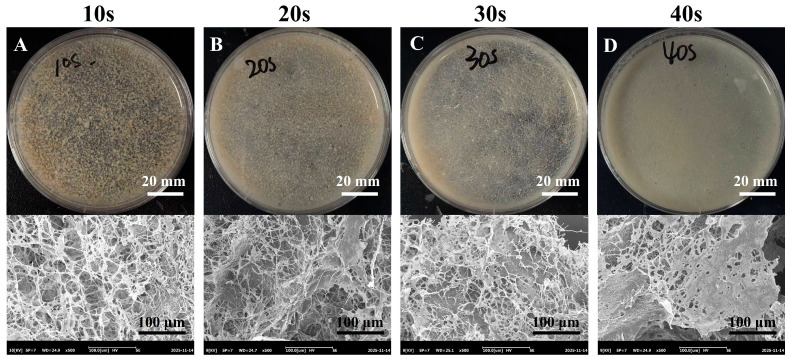
Morphology of *S. rugosoannulata* mycelium after different fragmentation times. (**A**–**D**) Macroscopic photographs and SEM images (at 500× magnification) of mycelial suspensions fragmented for 10, 20, 30, and 40 s.

The cultivated mycelium initially exhibited a pellet-like morphology. Because this spherical morphology is unsuitable for extrusion-based 3D printing, appropriate fragmentation is required to improve dispersion and meet the requirements for extrusion printing. As shown in Figure 1, both the macroscopic morphology and microscopic fibrous structure of *S. rugosoannulata* mycelium changed markedly with increasing fragmentation time. After 10 s of fragmentation, the mycelium remained mainly as large aggregates, resulting in a clearly granular suspension. SEM images showed tightly entangled hyphae forming a dense network structure. This indicated that the applied shear was insufficient to dissociate the mycelial aggregates effectively, leading to poor dispersion of the system.

When the fragmentation time was extended to 20 and 30 s, the dispersion state of the mycelium improved substantially. The suspension became more homogeneous, and most visible granularity disappeared. SEM observations showed that the mycelial fibers were well dispersed and formed a relatively loose but continuous fibrous network. These results suggest that moderate mechanical fragmentation effectively disentangles the mycelial aggregates while preserving the integrity of the filamentous structure. This dispersed fibrous network is beneficial for constructing a stable composite ink, as it can act as a physical scaffold and contribute to shape retention during extrusion-based 3D printing [28].

However, when the fragmentation time was further increased to 40 s, the mycelium showed signs of over-fragmentation. The SEM images revealed extensive breakage of mycelial fibers, accompanied by partial collapse and aggregation of the original fibrous network. The suspension also became more paste-like, indicating that excessive shear disrupted the structural integrity of the mycelial fibers. Such over-fragmentation may weaken the supporting function of the mycelial network and reduce its contribution to the formation of a stable 3D-printable gel system. Overall, the fragmentation time had a pronounced effect on the dispersion state and structural integrity of *S. rugosoannulata* mycelium. A fragmentation time of 20 s was considered suitable for balancing aggregate dissociation and fiber preservation. Therefore, mycelium fragmented within 20 s was selected for the subsequent preparation of mycelium–PPI–CUR composite inks.

### 3.2. 3D Printability of Composite Inks

**Figure 2:**
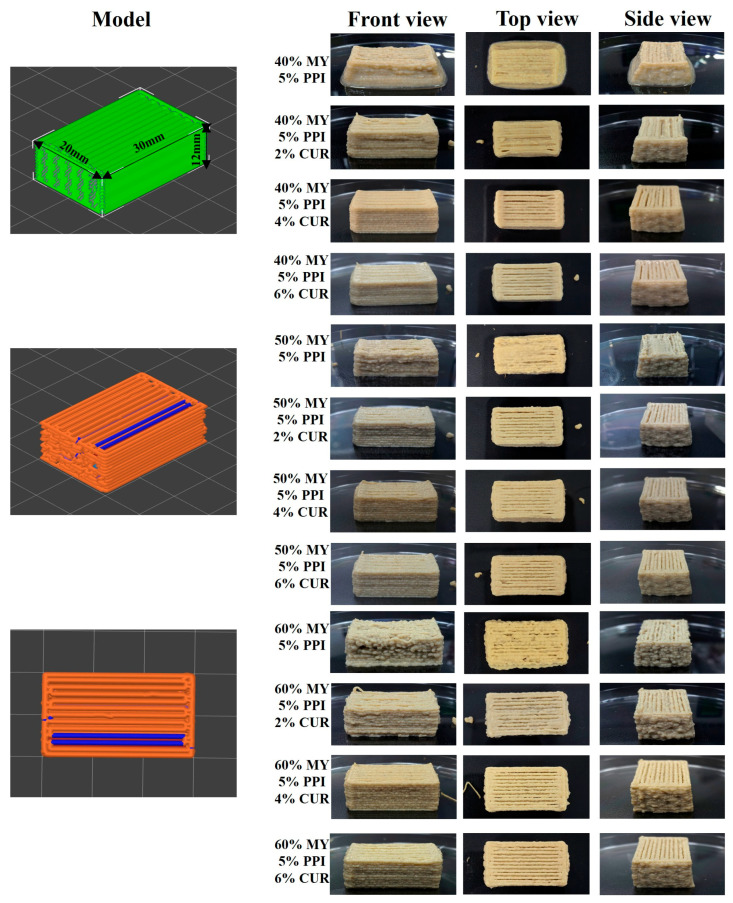
Visual appearance of 3D-printed mycelium–PPI–CUR composite inks with different mycelium and curdlan concentrations. MY represents mycelium.

The macroscopic appearance of the printed samples provides a direct visual evaluation of the printability of different formulations. As shown in Figure 2, the CUR-free control groups exhibited poor overall printability. The 40% mycelium control group showed severe structural collapse, edge slumping, and overall deformation, and it failed to maintain the cuboid geometry of the designed model. In addition, filament breakage and discontinuous extrusion occurred during printing. Although the 50% mycelium and 60% mycelium control groups did not collapse completely, they showed irregular filament stacking and disordered printing paths, indicating that the mycelium–PPI system alone was insufficient to meet the basic requirements for extrusion-based 3D printing.

When the CUR concentration was increased to 2%, the printability of all formulations improved, as indicated by more regular filament deposition and better visual quality. This improvement may be attributed to the thickening effects of CUR, which enhanced the shape retention of the composite inks [29]. With further increases in CUR concentration, the printability of the samples further improved, particularly in the 40% mycelium and 50% mycelium groups. When the CUR concentration reached 4% or higher, these two groups effectively reproduced the designed geometry, with neat edges, clear infill patterns, and improved shape fidelity. In contrast, the 60% mycelium group showed a different trend. Because of its high mycelium content, the CUR-free formulation exhibited uneven filament extrusion and was prone to nozzle clogging. After the CUR addition, the printing paths became more regular, and print accuracy improved. However, the combined thickening effects of high mycelium content and CUR resulted in excessive viscosity, which reduced effective extrusion continuity and produced a rough, granular surface. Therefore, although a high mycelium level contributed to structural support, excessive viscosity limited the overall printing performance.

**Figure 3:**
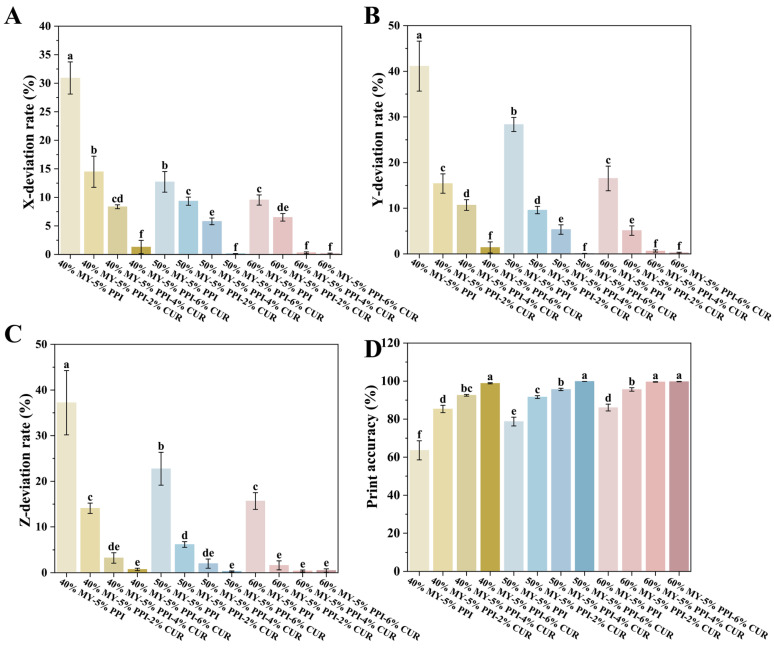
Dimensional deviation rate and print accuracy of mycelium–PPI–CUR composite inks with different mycelium and curdlan concentrations. (**A**) *X*-deviation rate; (**B**) *Y*-deviationsrate; (**C**) *Z*-deviation rate; (**D**) print accuracy. MY represents mycelium. Different lowercase letters indicate significant differences among groups.

The dimensional deviation rate and overall print accuracy further confirmed the visual observations described above. As shown in Figure 3, the CUR-free control groups showed larger dimensional deviations and lower print accuracy. In particular, the 40% mycelium control group showed deviations rate of 30.91% and 41.13% along the *X*- and *Y*-axes, respectively, and a *Z*-deviation rate of 37.22%. Its overall print accuracy was only 63.58%, indicating poor shape stability.

As the mycelium content increased from 40% to 60% in the CUR-free formulations, the dimensional deviation rate decreased. The *X*-deviation rate decreased from 30.91% to 9.54%, the *Y*-deviation rate decreased from 41.13% to 16.53%, and the *Z*-deviation rate decreased from 37.22% to 15.67%. Accordingly, the print accuracy increased from 63.58% to 86.09%. These results indicate that increasing mycelium enhanced the supporting capacity and dimensional retention of the printed structure to some extent. However, the printing paths remained irregular, preventing stable and high-fidelity printing. This result was consistent with the visual observations in Figure 2.

As the CUR concentration increased from 0% to 6%, the print accuracy of all formulations gradually increased. After the addition of 2% CUR, the print accuracy of all groups exceeded 85%. When the CUR concentration was increased to 4%, the printing accuracies of the 40% mycelium and 50% mycelium groups increased to 92.57% and 95.63%, respectively, while that of the 60% mycelium group approached 99.56%. At 6% CUR, the print accuracy of all groups exceeded 98%, with the 50% mycelium–5% PPI–6% CUR formulation showing the highest accuracy of 99.84%.

Nevertheless, although the 60% mycelium formulations with 4% and 6% CUR showed high print accuracy, they still exhibited excessive viscosity, reduced printing speed, and surface roughness. Therefore, their overall printing performance was inferior to that of the optimized 40% mycelium and 50% mycelium formulations. Considering macroscopic appearance, print accuracy, extrusion behavior, and processing adaptability, the 50% mycelium formulation provided the best balance between structural support and extrusion fluidity. Therefore, 50% mycelium was selected as the optimal mycelium level for subsequent experiments.

### 3.3. Rheological Properties of Mycelium–PPI–CUR Composite Inks

**Figure 4:**
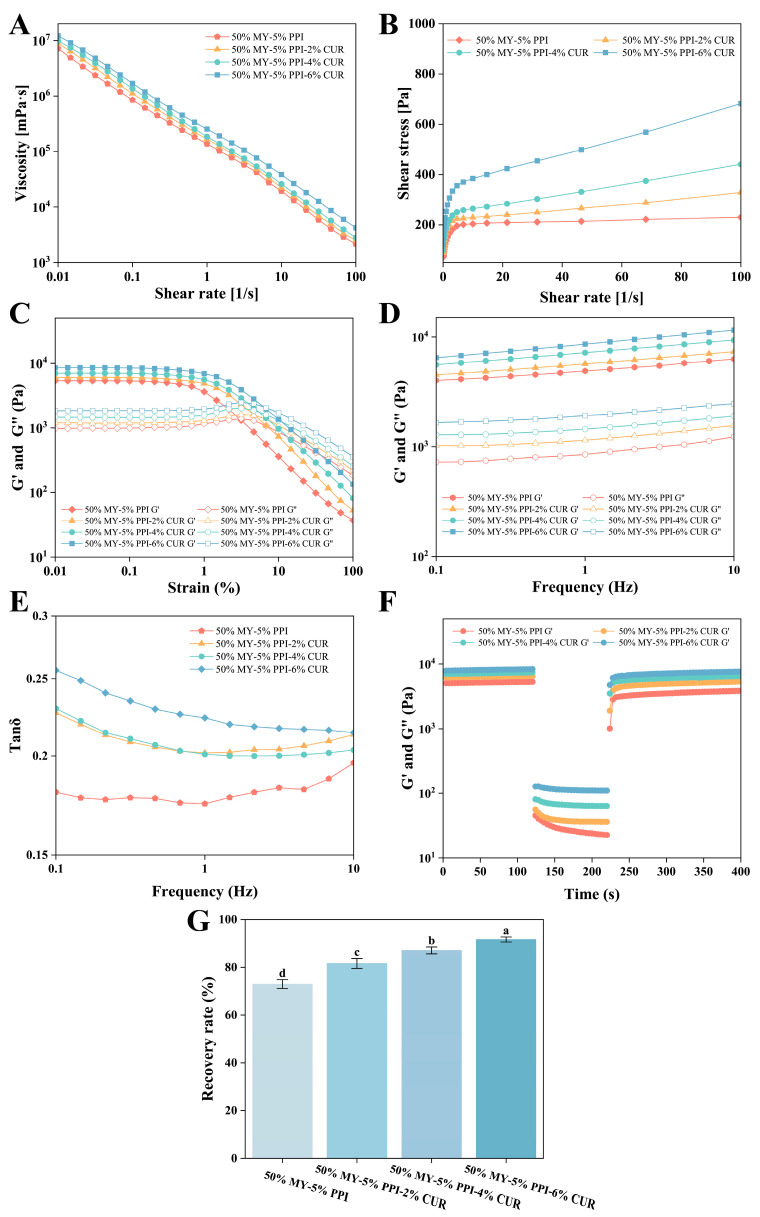
Rheological properties of mycelium–PPI–CUR composite inks with different CUR concentrations. (**A**) Shear viscosity; (**B**) Shear stress; (**C**) Amplitude sweep; (**D**) Frequency sweep; (**E**) Tan δ; (**F**) 3ITT; (**G**) Recovery rate. MY represents mycelium. Different lowercase letters indicate significant differences among groups.

The success of extrusion-based 3D food printing largely depends on the rheological properties of printing inks, particularly their flow behavior during extrusion and their structural recovery after deposition [30]. As shown in Figure 4A, the apparent viscosity of all composite inks decreased as the shear rate increased from 0.01 to 100 s^−1^, indicating typical shear-thinning behavior. This behavior is favorable for extrusion-based 3D printing because the ink can flow smoothly under the high shear generated in the nozzle while maintaining sufficient viscosity after deposition [31].

The addition of CUR increased the viscosity of the composite inks. At the same shear rate, the viscosity increased gradually with increasing CUR concentration. The control ink without CUR, namely 50% mycelium–5% PPI, consistently exhibited the lowest viscosity, whereas the 6% CUR concentration ink showed the highest viscosity. This result indicates that CUR enhanced the consistency and cohesiveness of the mycelium–PPI system. The increased viscosity may be attributed to the hydration and chain entanglement of CUR, as well as possible interactions among CUR, mycelial fibers, and PPI molecules. These interactions could contribute to the formation of a denser composite network, thereby improving the resistance of the system to flow deformation.

The shear stress curves further confirmed the strengthening effect of CUR on the composite inks. As shown in Figure 4B, the shear stress of all samples increased continuously with increasing shear rate. At a given shear rate, the shear stress increased with increasing CUR concentration. At a shear rate of 100 s^−1^, the shear stress of the CUR-free control ink was approximately 228 Pa, whereas that of the ink containing 6% CUR reached approximately 680 Pa. This indicates that a higher external force was required to drive the flow of CUR-containing inks, suggesting that CUR reinforced the internal network structure of the composite system.

The strain sweep results are shown in Figure 4C. In the low-strain region, the G′ of all inks was higher than the G″, indicating that the inks exhibited elastic-dominated and solid-like behavior [32]. With increasing CUR concentration, the G′ increased, and the LVR became broader, indicating enhanced network strength and improved resistance to deformation. These properties are beneficial for maintaining filament integrity and supporting layer-by-layer stacking during 3D printing.

The frequency sweep results further demonstrated the gel-like characteristics of the composite inks. As shown in Figure 4D, G′ was consistently higher than G″ over the entire frequency range of 0.1–10 Hz for all formulations, confirming that the inks were dominated by elastic behavior. Both G′ and G″ increased with increasing CUR concentration, suggesting that CUR promoted the formation of a stronger and more stable network. This reinforced network may be attributed to the structural features of CUR, a linear β-1,3-glucan rich in hydroxyl groups [14]. Specifically, higher CUR concentrations increase polymer chain density. This leads to more extensive physical entanglements and stronger intermolecular interactions among CUR, PPI, and the mycelial fibers. Tan δ, defined as the ratio of G″ to G′, reflects the viscoelastic behavior of the inks. As shown in Figure 4E, all inks exhibited solid-like characteristics with tan δ < 1, indicating elasticity-dominated behavior. Although tan δ increased slightly with CUR concentration, all samples remained in the elasticity-dominant regime (tan δ < 1), indicating that the inks still possessed sufficient structural integrity for shape retention after deposition [33].

The structural recovery behavior of the inks was evaluated using 3ITT, which simulates the structural breakdown during extrusion and recovery after deposition [34]. As shown in Figure 4F, G′ decreased sharply during the high-strain interval, indicating disruption of the gel network under shear. When the strain was reduced again, G′ rapidly recovered, demonstrating the self-recovery ability of the inks. The recovery rate increased with increasing CUR concentration, from 72.98% for the control ink to 81.61%, 87.02%, and 91.67% for the inks containing 2%, 4%, and 6% CUR, respectively (Figure 4G). The improved recovery behavior indicates that CUR facilitated network reconstruction after shear disruption, which is essential for maintaining shape fidelity after extrusion.

Overall, the rheological results demonstrated that CUR effectively improved the viscosity, viscoelasticity, and structure recovery of the mycelium–PPI composite inks. These rheological improvements explain the enhanced extrusion continuity, print accuracy, and shape retention observed in the printing experiments.

### 3.4. WHC of Composite Inks

**Figure 5:**
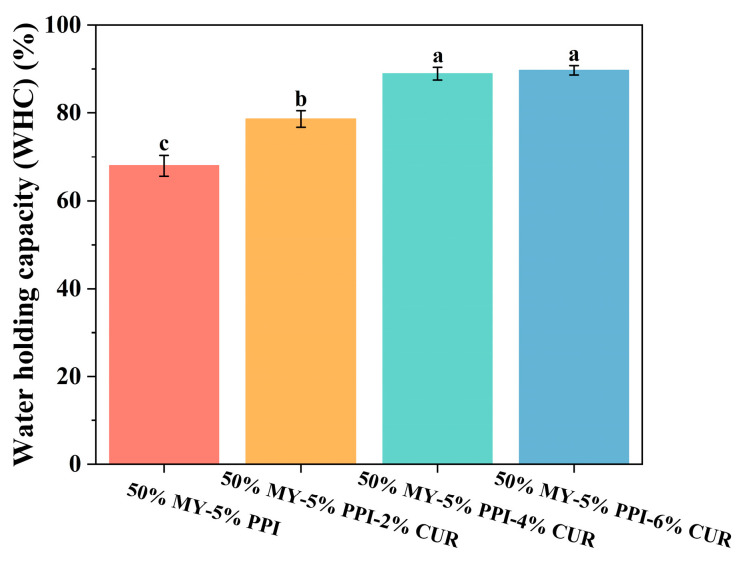
Water-holding capacity of mycelium–PPI–CUR composite gels with different CUR concentrations. MY represents mycelium. Different lowercase letters indicate significant differences among groups.

WHC is an important indicator of the structural stability of gel systems and strongly influences extrusion-based 3D printability. Insufficient WHC may cause printing defects such as water exudation, filament collapse, and dimensional deviation during extrusion and deposition. The effects of CUR concentration on the WHC of the composite gels are shown in Figure 5.

Overall, the WHC of the composite gels increased with increasing CUR concentration. The 50% mycelium–5% PPI group showed the lowest WHC of 67.98%, indicating that the mycelium–PPI binary system had limited ability to retain water. After CUR incorporation, the WHC increased significantly from 67.98% to 88.92% as the CUR concentration increased from 0% to 4%. This improvement may be attributed to the water-binding and network-forming abilities of CUR. As a hydrophilic β-1,3-glucan, CUR can retain water through hydrogen bonding [35]. In addition, CUR may interact with PPI and mycelial components through hydrogen bonding and molecular entanglement, thereby strengthening the gel network and improving water retention.

There was no significant difference in WHC between 4% CUR and 6% CUR, indicating that the WHC tended to plateau at higher CUR levels. This suggests that the CUR concentrations above 4% had a limited effect on WHC. Therefore, CUR effectively improved the WHC of mycelium–PPI composite inks, which contributed to enhanced dimensional stability during 3D printing.

### 3.5. Water Distribution in Composite Inks

LF-NMR was used to characterize the water distribution in the composite inks based on the transverse relaxation time (T_2_) of hydrogen protons [36]. Generally, a shorter T_2_ indicates stronger interactions between water molecules and gel macromolecules, whereas a longer T_2_ suggests greater water mobility. The physical state and spatial distribution of water within the internal structure of the gel directly dictate the stability of the network structure, rheological properties, and 3D printability of the system [37].

**Figure 6:**
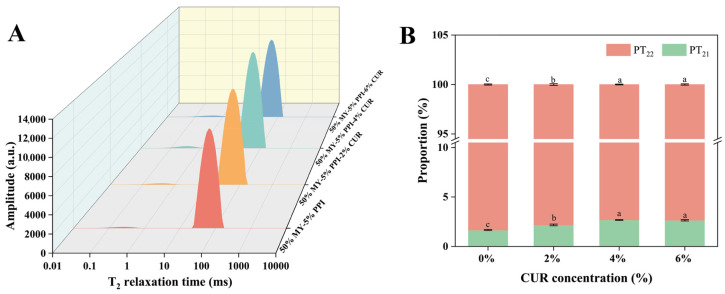
Water status and distribution of mycelium–PPI composite inks with different CUR concentrations. (**A**) T_2_ relaxation time distribution; (**B**) Relative peak area proportions of different water populations. MY represents mycelium. Different lowercase letters indicate significant differences among groups.

As shown in Figure 6A, according to the T_2_ relaxation time, two major water populations were identified: bound water with a short relaxation time of 0.01–10 ms, and immobilized water with a longer relaxation time of 10–100 ms; no distinct free water peak was observed. As shown in Figure 6B, immobilized water accounted for more than 97% of the total peak area in all formulations. With increasing CUR concentration, the proportion of bound water increased slightly and reached a maximum of 2.68% in the 4% CUR group, while the 6% CUR group showed a comparable value. This suggests that CUR promoted water redistribution within the composite ink by forming a denser three-dimensional network, thereby converting part of the immobilized water into bound water. Consequently, the WHC and structural stability of the composite inks were improved.

Overall, the LF-NMR results confirmed that CUR effectively modulated the water state in the mycelium–PPI composite inks. Importantly, these microscopic changes in water distribution correlate with the macroscopic rheological properties discussed in Section 3.3. The increased proportion of bound water indicates a tighter, extensively cross-linked network. This tightly bound state restricts overall water mobility, thereby reducing the internal lubricating effect between polymer chains. Consequently, the system exhibits stronger resistance to deformation (resulting in a higher G′ and elevated shear stress) and a faster structural reconstruction upon the removal of shear forces (yielding a higher thixotropic recovery rate).

### 3.6. FTIR

**Figure 7:**
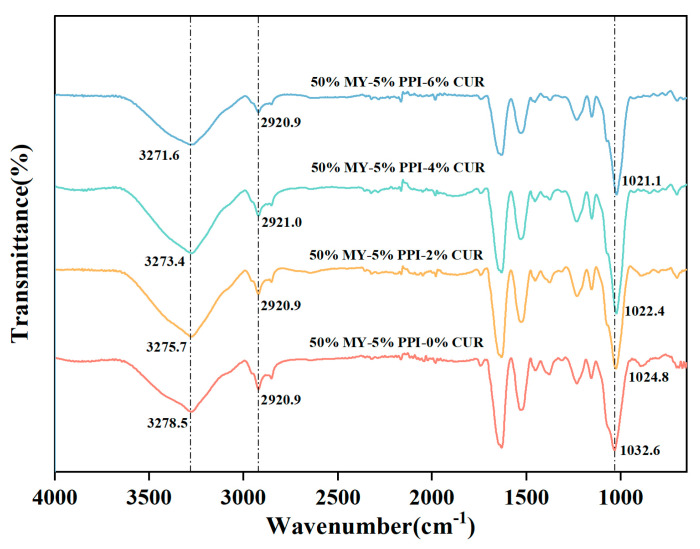
FTIR spectra of mycelium–PPI–CUR composite inks with different CUR concentrations. MY represents mycelium.

FTIR spectroscopy was performed to clarify the molecular interactions in the mycelium–PPI composite inks with different CUR concentrations [38]. As shown in Figure 7, all samples presented a broad band at 3200–3400 cm^−1^, which is attributed to the stretching vibrations of O–H and N–H groups [39]. The peak near 2920 cm^−1^ represents asymmetric C-H stretching vibrations of methylene (CH2) groups [40]. With increasing CUR addition, the absorption peak at the 3200–3400 cm^−1^ region shifted from 3278.5 cm^−1^ (0% CUR) to 3275.7, 3273.4, and 3271.6 cm^−1^ for the 2%, 4%, and 6% CUR groups, respectively. The shift toward lower wavenumbers indicates strengthened hydrogen-bonding interactions [41]. This result suggests that CUR interacted with mycelial components and PPI through intermolecular hydrogen bonding, thereby strengthening the molecular association within the composite matrix. Such enhanced hydrogen bonding promoted the formation of a more cohesive and compact network structure. In addition, the peak in the 1000–1150 cm^−1^ region was attributed to C–O and C–O–C stretching vibrations of polysaccharides, reflecting the contribution of carbohydrate components, including CUR, to the composite network [42]. No obvious new peaks were detected after CUR incorporation, implying that the interaction among components was dominated by non-covalent forces rather than the formation of new covalent bonds.

### 3.7. Thermal Properties

**Figure 8:**
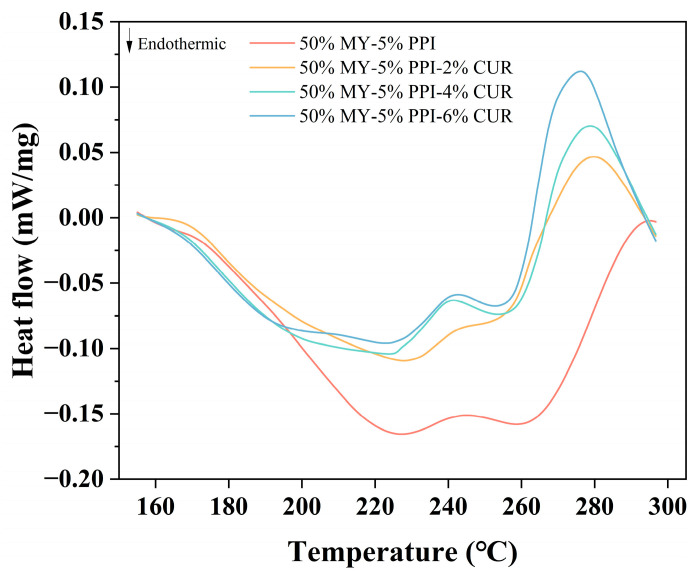
DSC thermograms of mycelium–PPI–CUR composite inks with different CUR concentrations. The downward arrow indicates the endothermic direction. MY represents mycelium.

DSC was used to evaluate the thermal transition behavior of the mycelium–PPI–CUR composite inks [43]. As shown in Figure 8, all samples exhibited an endothermic region between approximately 160 and 270 °C, which may be related to the dissociation of intermolecular interactions, protein conformational changes, and thermal rearrangement of polysaccharide chains within the composite network [44]. Given the multicomponent nature of the system, this region likely reflects overlapping thermal events rather than a single transition.

The CUR-free control group exhibited the greatest endothermic intensity. In contrast, the endothermic intensity and the heat flow gradually decreased with increasing CUR addition. The reduced endothermic intensity suggests that less heat was consumed by structural dissociation after CUR incorporation [45]. A possible explanation is that CUR enhanced intermolecular interactions within the composite matrix, thereby promoting the formation of a more compact and stable gel network [14]. As a result, the number of free or weakly bound structures that could be readily disrupted upon heating was reduced, while some labile associations may have been replaced by a more stable composite structure. This interpretation is supported by the FT-IR results, where the hydrogen bond-related band shifted toward lower wavenumbers with increasing CUR content, indicating strengthened hydrogen bond interactions.

Overall, CUR incorporation changed the thermal transition behavior of the mycelium–PPI composite inks, reduced the intensity of the endothermic peaks, and improved the structural resistance of the network against heat-induced dissociation. These results are consistent with the rheological findings and the observed increase in WHC.

### 3.8. Quality Assessment of 3D-Printed Meat Analogs

**Figure 9:**
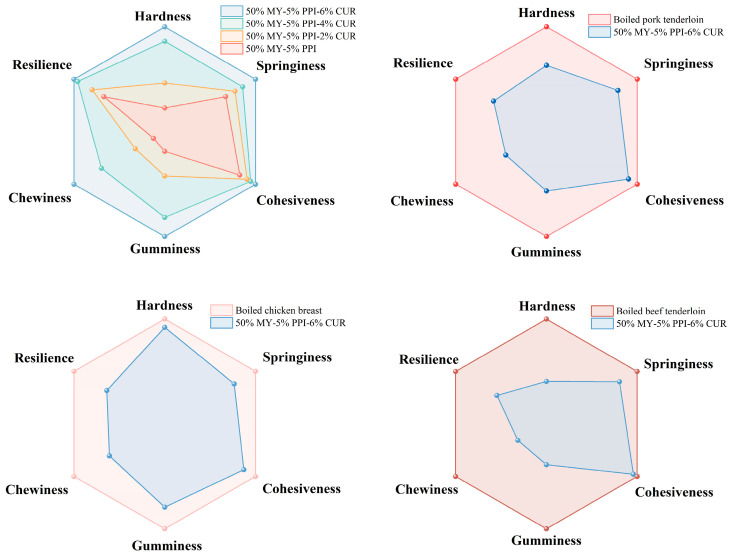
Radar charts of textural properties of mycelium-based meat analogs and boiled meats. MY represents mycelium.

Textural properties are key indicators for evaluating the structural integrity, mouthfeel, and potential consumer acceptability of mycelium-based meat analogs. The textural properties of the printed and steamed meat analogs are summarized in Table 2 and Figure 9. The incorporation of CUR significantly improved the textural properties of the mycelium-based meat analogs, and all measured TPA parameters increased progressively with increasing CUR concentration.

The control sample without CUR (50% mycelium-5% PPI) showed the weakest textural properties. Its hardness was 1020.34 g, and its springiness, cohesiveness, gumminess, chewiness, and resilience were 0.435, 0.396, 404.56, 175.26, and 0.131, respectively. These results indicate that the CUR-free mycelium–PPI matrix formed a relatively weak and loose gel network with limited resistance to deformation. As the CUR concentration increased from 2% to 6%, the textural parameters of the meat analogs increased significantly. Specifically, hardness increased from 2101.23 g to 4511.06 g; springiness increased from 0.501 to 0.648; cohesiveness increased from 0.434 to 0.479; gumminess increased from 911.29 to 2160.13; chewiness increased from 457.09 to 1406.00; and resilience increased from 0.156 to 0.195. The improvement in texture can be attributed to the increased CUR concentration and its thermally induced gelation, which collectively reinforce the mycelium–PPI network. During steaming, CUR forms a thermo-irreversible gel network, which can interpenetrate with the protein–mycelial fiber matrix and strengthen the overall gel structure.

A radar chart was used to compare the textural similarity between the optimized meat analog and conventional cooked meats. The meat analogs printed using 50% mycelium–5% PPI–6% CUR were compared with boiled chicken breast, pork tenderloin, and beef tenderloin using texture radar charts, as shown in Figure 9. It showed the highest textural similarity to boiled chicken breast, with a radar-chart overlap of 72.3%, followed by pork tenderloin (49.2%) and beef tenderloin (30.3%). These results indicate that the mycelium-based meat analog more closely resembles poultry muscle, particularly chicken breast, than mammalian muscle tissues. This could be attributed to the fact that chicken breast has finer muscle fibers and a relatively tender texture, which better matches the mechanical properties of the mycelial network. In contrast, beef and pork tenderloin possess thicker muscle fibers, rendering them significantly harder and chewier than the meat analog, thereby resulting in a lower textural similarity.

## 4. Conclusions

In this study, *S. rugosoannulata* mycelium, PPI, and CUR were combined to develop extrusion-based 3D-printable inks for meat analogs. Mechanical fragmentation for 20 s effectively dispersed the mycelial aggregates while preserving the filamentous network essential for ink preparation. Among the formulations, 50% mycelium provided the optimal balance between structural support and extrusion fluidity. Notably, the incorporation of CUR significantly enhanced the rheological properties, water retention, and printability of the composite system, with the 50% mycelium–5% PPI–6% CUR formulation achieving a peak print accuracy of 99.84%. Mechanistic insights from LF-NMR, FT-IR, and DSC analyses demonstrated that CUR strengthened intermolecular hydrogen bonding, promoted the transition of immobilized water to bound water, and elevated the thermal stability of the gel network. Following steaming, the texture of the printed analogs improved progressively with increasing CUR concentration. Ultimately, the optimized formulation achieved a 72.3% textural similarity to boiled chicken breast based on radar-chart analysis. This study provides a practical strategy for developing sustainable fungal protein-based meat analogs using extrusion-based 3D printing. Despite the successful optimization of the physicochemical and textural properties of these mycelium-based meat analogs, translating these laboratory-scale findings into industrial applications faces several limitations. First, scaling up the 3D printing process remains a significant engineering challenge. Second, minor fluctuations in fermentation parameters (e.g., pH, temperature, and nutrients) can alter mycelial properties, leading to batch-to-batch variability. Additionally, downstream processing steps like biomass recovery, washing, and fragmentation are costly and energy-intensive, which could impact the overall economic viability of large-scale production. To achieve ultimate commercial success, future research must overcome these technical hurdles while comprehensively evaluating the in vitro digestibility, digestive kinetics, stability of 3D-printed structures during longer storage or reheating conditions and consumer sensory acceptance of the final products.

## Figures and Tables

**Table 1:** Formulations of mycelium–PPI–CUR composite inks.

Sample	CUR (%)	Mycelium (%)	PPI (%)	Deionized Water (%)
40% MY-5% PPI	0	40	5	55
50% MY-5% PPI	0	50	5	45
60% MY-5% PPI	0	60	5	35
40% MY-5% PPI-2% CUR	2	40	5	53
50% MY-5% PPI-2% CUR	2	50	5	43
60% MY-5% PPI-2% CUR	2	60	5	33
40% MY-5% PPI-4% CUR	4	40	5	51
50% MY-5% PPI-4% CUR	4	50	5	41
60% MY-5% PPI-4% CUR	4	60	5	31
40% MY-5% PPI-6% CUR	6	40	5	49
50% MY-5% PPI-6% CUR	6	50	5	39
60% MY-5% PPI-6% CUR	6	60	5	29

Note: All components are expressed as weight percentages, *w*/*w*. MY represents mycelium.

**Table 2:** Textural properties of 3D-printed mycelium-based meat analogs with varying CUR concentrations.

Sample	Hardness (g)	Springiness	Cohesiveness	Gumminess	Chewiness	Resilience
50% MY-5% PPI	1020.34 ± 39.74 ^g^	0.435 ± 0.010 ^g^	0.396 ± 0.013 ^g^	404.56 ± 12.00 ^g^	175.26 ± 1.73 ^g^	0.131 ± 0.004 ^g^
50% MY-5% PPI-2% CUR	2101.23 ± 48.30 ^f^	0.501 ± 0.013 ^f^	0.434 ± 0.010 ^f^	911.29 ± 22.84 ^f^	457.09 ± 25.82 ^f^	0.156 ± 0.006 ^f^
50% MY-5% PPI-4% CUR	3894.11 ± 28.12 ^e^	0.556 ± 0.014 ^e^	0.453 ± 0.007 ^e^	1764.80 ± 62.23 ^e^	980.02 ± 64.32 ^e^	0.187 ± 0.003 ^e^
50% MY-5% PPI-6% CUR	4511.06 ± 41.59 ^d^	0.648 ± 0.011 ^d^	0.479 ± 0.005 ^d^	2160.13 ± 82.54 ^d^	1406.00 ± 55.63 ^d^	0.195 ± 0.003 ^d^
Boiled Chicken Breast	4900.51 ± 123.76 ^c^	0.850 ± 0.010 ^a^	0.550 ± 0.010 ^a^	2718.91 ± 114.63 ^c^	2305.72 ± 132.84 ^c^	0.310 ± 0.010 ^c^
Boiled Pork Tenderloin	7133.01 ± 96.91 ^b^	0.820 ± 0.010 ^b^	0.530 ± 0.010 ^b^	3813.15 ± 23.70 ^b^	3140.85 ± 47.55 ^b^	0.330 ± 0.010 ^b^
Boiled Beef tenderloin	11,100.29 ± 436.60 ^a^	0.800 ± 0.010 ^c^	0.500 ± 0.010 ^c^	5559.08 ± 162.19 ^a^	4474.15 ± 185.00 ^a^	0.360 ± 0.010 ^a^

Note: Data are presented as mean ± standard deviation, *n* = 3. Different lowercase letters within the same column indicate significant differences among groups (*p* < 0.05). MY represents mycelium.

## Data Availability

The data presented in this study are available on request from the corresponding author.
